# Sharp rises in FGF23 and hypophosphatemia after intravenous iron administration do not cause myocardial damage

**DOI:** 10.1007/s00392-020-01630-z

**Published:** 2020-03-25

**Authors:** Vincent Brandenburg, Gunnar H. Heine, Nikolaus Marx, Robert Stöhr

**Affiliations:** 1 Rhein-Maas-Klinikum, Würselen, Germany; 2 Department of Internal Medicine I, University Hospital RWTH Aachen, Pauwelstrasse 30, 52074 Aachen, Germany; 3 AGAPLESION MARKUS KRANKENHAUS, Frankfurt am Main, Germany

Sirs:

Current guidelines recommend intravenous iron replenishment (specifically by ferric carboxymaltose [FCM]) in iron-deficient patients with heart failure and reduced ejection fraction (HFrEF). We have previously shown that the administration of FCM in HFrEF patients with normal kidney function leads to an acute increase in circulating intact FGF23 (iFGF23) with a significant and concurrent hypophosphataemia that persists over the span of several weeks [IRON-TURTLE trial (NCT03079518)] [1]. Numerous human cohort studies have postulated an independent association between elevated serum levels of FGF23 and an increased risk for cardiovascular disease and mortality [2]. Current hypotheses suggest that this association is caused by the direct effects of FGF23 on inducing myocardial hypertrophy [3]. Thus, FCM-induced increases in FGF23, paralleled by relevant hypophosphatemia, may compound to cause (additional) myocardial damage in patients with heart failure before the beneficial long-term effects of iron replenishment occur [4]. Furthermore, data suggest, that FGF23 may influence inflammation by directly stimulating the production of inflammatory markers from the liver [5, 6]. Still, to date, no data exist on such potentially harmful short-term effect of FCM in patients.

The IRON-TURTLE trial included 23 patients with iron deficiency and HFrEF, all of whom received 1000 mg FCM. As reported before, FCM infusion significantly increased iFGF23 levels up to 11-fold within one day, returning back to baseline levels after four weeks, in a subgroup of 11 patients with normal renal function (eGFR 74.8 ± 12.8 ml/min/m^2^). In parallel to this iFGF23 rise, 60% of these patients developed significant hypophosphatemia (mean decrease 28% ± 16% from baseline) [1]. Thus, FCM-induced increases in FGF23, paralleled by a relevant hypophosphataemia, may compound to cause (additional) myocardial damage in patients with heart failure constituting a previously underestimated (transient) post-infusion risk in these patients before the beneficial long-term effects of iron replenishment occur.

We, therefore, measured levels of various established biomarkers of myocardial damage and heart failure [NT-proBNP, MR-proANP, Copeptin-proAVP, Big Endothelin and MR-proadrenomedulin (MR-proADM)] [7, 8] as well as markers of renal function (GFR, Cystatin C, NGAL and KIM1) and markers of inflammation (TNF-alpha, Interleukin-6 (IL-6) and C-reactive protein (CRP)) in the aforementioned subgroup of patients with normal renal function over the course of 4 weeks. The log-transformed values of all biomarkers were used to model each marker in linear mixed models with log-transformed iFGF23 as an independent continuous variable and visits as fixed factor. Random intercepts for subjects were included in the models. Models were estimated using the restricted maximum likelihood method, and 95% profile confidence intervals were calculated for the coefficient estimates.

As depicted in Table 1, FCM infusion did not lead to any significant longitudinal fluctuations in cardiac and renal injury markers to parallel the changes in FGF23 and phosphate. There was a small association between rises in FGF23 and a decrease in TNF-alpha and IL-6. However, the effects seen were small.

**Table 1:** Serum parameters over time

	Baseline	Day 1	Day 7	Day 14	Day 28	Rel. effect (95% CI)
iFGF23 (pg/ml)	17.3 ± 11.0	178.9 ± 131.6***	126.5 ± 80.5***	65.9 ± 56.0**	24.5 ± 23.4	
Phosphate (mmol/l)	1.09 ± 0.21	1.06 ± 0.21	0.76 ± 0.20*	0.69 ± 0.21**	0.88 ± 0.2	
NT-proBNP (pg/ml)	4829 ± 9573	3943 ± 7839	4708 ± 9322	3834 ± 9610	2271 ± 4856	0.963 (0.873–1.061)
Big Endothelin (nmol/l)	0.79 ± 0.32	0.92 ± 0.56	0.66 ± 0.27	0.79 ± 0.28	0.85 ± 0.29	0.961 (0.797–1.158)
MR-proANP (nmol/ml)	6.34 ± 4.21	6.28 ± 5.55	6.18 ± 4.38	5.76 ± 4.18	5.91 ± 3.95	1.015 (0.929–1.108)
Copeptin-proAVP (pmol/l)	14.07 ± 19.45	19.12 ± 23.25	10.76 ± 14.95	12.21 ± 17.21	14.67 ± 23.7	1.009 (0.884–1.152)
MR-proADM (nmol/l)	0.83 ± 0.27	0.86 ± 0.65	0.79 ± 0.34	0.82 ± 0.34	0.85 ± 0.28	1.011 (0.909–1.123)
GFR (ml/min/m^2^)	74.7 ± 15.9	74.41 ± 17.23	77.3 ± 23.4	81.5 ± 20	80.6 ± 22.6	0.968 (0.912–1.027)
NGAL (pg/ml)	107.982 ± 52.684	144.358 ± 73.936	112.302 ± 63.389	103.499 ± 101.550	104.953 ± 79.767	0.943 (0.833–1.068)
KIM-1 (pg/ml)	466.4 ± 645.1	427.1 ± 545.7	511.7 ± 614,4	530.7 ± 531.4	320.3 ± 401.5	1.386 (0.987–1.946)
Cystatin C mg/l	1.16 ± 0.48	1.16 ± 0.42	1.21 ± 0.45	1.06 ± 0.30	1.14 ± 0.38	1.015 (0.977–1.055)
TNF-alpha (ng/ml)	7.8 ± 2.2	9.2 ± 2.7	7.9 ± 2.4	10.4 ± 12.2	8.6 ± 3.6	0.927 (0.870–0.987)*
IL-6 (pg/ml)	16.8 ± 15.4	18.1 ± 17.1	18.3 ± 30.3	18.5 ± 37.2	13.4 ± 18.9	0.850 (0.739–0.977)*
hs-CRP	10.1 ± 15.7	10.5 ± 16.9	16.3 ± 39.9	22.3 ± 39.9	11.4 ± 28.1	0.989 (0.808–1.209)

***p < 0.001, **p < 0.01, *p < 0.05

Our data obtained from a selected HFrEF cohort are of particular interest because previous experimental work has postulated a direct negative effect of FGF23 on the myocardium [3]. Despite an 11-fold relative rise in iFGF23 induced by FCM infusion, a level which by far exceeds the threshold levels of iFGF23 for which an increase of mortality was proven in associative human cohort studies [9], we do not see any adverse effect in our broad panel of cardiac and renal damage biomarkers. While one may argue that these short-term effects do not reflect the long-term exposure to FGF23 that renal failure and dialysis patients experience, previous experimental work has shown that increasing the level of FGF23 by just fourfold can lead to the development of acute myocardial damage and dysfunction after a short duration of 5 days [3]. Interestingly, our data on inflammatory markers are in stark contradiction to previously published mouse data which had shown that increases in FGF23 induce the hepatic production of CRP and IL-6 through calcineurin-signalling, also in a short time span of 5 days [5].

Our data suggest that FCM does not induce transient myocardial stress and damage despite raising plasma FGF23 to levels that far exceed those shown to cause cardiac damage in previous experimental models. Similarly, our data challenge the current theory, that increases in FGF23 can directly influence hepatic inflammation.

Thus, the long-term beneficial effects of FCM administration in HFrEF patients which have been reported before [4] are not counterbalanced by acute, FGF-23 induced adverse cardiac effects. Hence, our clinical results on circulating biomarkers suggest that currently propagated rodent model data regarding myocardial damage and inflammatory stimulation induced by FGF23 cannot be transferred directly to the human situation. The biological meaning of FGF23-induced transient hypophosphatemia after FCM infusion in HFrEF patients remains to be determined, as does the effect of repetitive dosing.
